# Predictive Role of Tumor-Stroma Ratio for Survival of Patients With Non-Small Cell Lung Cancer: A Meta-Analysis

**DOI:** 10.3389/pore.2021.1610021

**Published:** 2022-01-21

**Authors:** Xuefeng Zhang, Hongfu Ma, Liang Zhang, Fenghuan Li

**Affiliations:** Department of Respiratory and Critical Care Medicine, Yantai Mountain Hospital, Yantai, China

**Keywords:** recurrence, survival, meta-analysis, non-small cell lung cancer, tumor-stroma ratio

## Abstract

**Background:** Role of tumor-stroma ratio (TSR) as a predictor of survival in patients with non-small cell lung cancer (NSCLC) remains not clear. A systematic review and meta-analysis was conducted to summarize current evidence for the role of TSR in NSCLC.

**Methods:** Relevant cohort studies were retrieved via search of Medline, Embase, and Web of Science databases. The data was combined with a random-effect model by incorporating the between-study heterogeneity. Specifically, subgroup and meta-regression analyses were performed to explore the association between TSR and survival in patients with squamous cell carcinoma (SCC) or adenocarcinoma (AC).

**Results:** Nine cohort studies with 2031 patients with NSCLC were eligible for the meta-analysis. Pooled results showed that compared to those stroma-poor tumor, patients with stroma rich NSCLC were associated with worse recurrence-free survival (RFS, hazard ratio [HR] = 1.52, 95% confidence interval [CI]: 1.07 to 2.16, *p* = 0.02) and overall survival (OS, HR = 1.48, 95% CI: 1.20 to 1.82, *p* < 0.001). Subgroup analyses showed that stroma-rich tumor may be associated with a worse survival of SCC (HR = 1.89 and 1.47 for PFS and OS), but a possibly favorable survival of AC (HR = 0.28 and 0.69 for PFS and OS). Results of meta-regression analysis also showed that higher proportion of patients with SCC was correlated with higher HRs for RFS (Coefficient = 0.012, *p* = 0.03) and OS (Coefficient = 0.014, *p* = 0.02) in the included patients, while higher proportion of patients with AC was correlated with lower HRs for RFS (Coefficient = −0.012, *p* = 0.03) and OS (Coefficient = −0.013, *p* = 0.04), respectively.

**Conclusion:** Tumor TSR could be used as a predictor of survival in patients with NSCLC. The relative proportion of patients with SCC/AC in the included NSCLC patients may be an important determinant for the association between TSR and survival in NSCLC. Stroma richness may be a predictor of poor survival in patients with lung SCC, but a predictor of better survival in patients with lung AC.

## Introduction

Among various solid tumors, lung cancer is a common malignancy and a leading cause of cancer-specific mortality [1]. The annual deaths related to lung cancer are reported to be more than 1.7 million all over the world [2]. Non-small cell lung cancer (NSCLC) is the most common category of lung cancer, which takes up for about 85% of the overall lung cancer patients [3]. Currently, despite a comprehensive treatment strategies including of surgery resection, chemotherapy, radiotherapy, immune therapy, and targeted therapy, the prognosis of NSCLC patients remain not satisfying [4, 5]. Clinically, identification of prognostic histological marker is still important for optimizing risk stratification and choosing of personalized treatment in patients with NSCLC [6, 7].

Changes of tumour microenvironment have been recognized as key determinants in the progression of the disease [8, 9]. Tumor stroma, which refers to complicated components of non-neoplastic cells such as fibroblasts, endothelial cells, and immune cells, as well as the extracellular protein matrix, have been confirmed to be actively involved in the processes of carcinogenesis and metastasis [10]. Interestingly, it has been shown that tumor-stroma ratio (TSR), an indicator for the amount of tumour-associated stroma at invasive tumor on traditional hematoxylin and eosin (H&E)-stained paraffin sections, may a predictor of poor prognosis in solid tumor, such as breast and colon cancers [11, 12]. Indeed, a previous meta-analysis showed that higher proportion of stroma in primary cancer tissue was associated with poor prognosis of the patients, although studies with various types of cancer were included and a site-specific association between TSR and survival in patients with solid tumor was suggested [13]. Role of tumor-stroma ratio (TSR) as a predictor of survival in patients with non-small cell lung cancer (NSCLC) remains inconsistent according to previous studies [14–22]. Patients with stroma-rich NSCLC were shown to have poor survival in some studies [14, 15, 17, 19, 20], but not in others [16, 18, 21, 22]. Accordingly, this systematic review and meta-analysis was conducted to summarize current evidence regarding the possible predictive role of TSR for survival in patients with NSCLC. In particular, previous studies suggested possible differences in immune host response and role of tumor immune microenvironment in lung squamous cell carcinoma (SCC) and AC (adenocarcinoma) [23–25], we also explored whether the potential association between TSR and survival outcomes is different in patients with lung SCC and AC.

## Methods

The instructions of MOOSE (Meta-analysis of Observational Studies in Epidemiology) [26] and Cochrane’s Handbook [27] were followed in the study.

### Literature Search

Studies were obtained by search of electronic databases of Medline, Embase, and Web of Science via the combined search terms: 1) “tumor-stroma” OR “tumour-stroma” OR “tumor stroma” OR “tumour stroma” OR “Glasgow tumor microenvironment score”; 2) “lung cancer”; and 3) “survival” OR “prognosis” OR “mortality” OR “recurrence” OR “recurrent” OR “death” OR “metastasis” OR “progression” OR “hazard ratio” OR “surgery” OR “operation” OR risk.” Only stuides in human was considered, and the publication language was limited to English or Chinese. The references of related original and review articles were further screened annually for possible studies. The literature search was finally performed on May 10, 2021.

### Study Selection

The inclusion criteria were: 1) cohort studies; 2) included patients with confirmed diagnosis of NSCLC; 3) evaluated the association between TSR and survival outcomes of the patients, including recurrence-free survival (RFS) and overall survival (OS); and 4) reported the hazard ratio (HR) for at least one of the above survival outcomes comparing between patients with stroma-rich (low TSR) and stroma-poor (high TSR) NSCLC. Reviews, preclinical studies, studies including patients with other cancers, studies that did not evaluate TSR, or studies without available outcome data were not included.

### Data Extracting and Quality Evaluation

The processes of database search, data extraction, and study quality assessment were independently and separately performed by two authors. If discrepancies occurred, discussion with the corresponding author was indicated. Following data were recorded into a predefined Excel form for data management: 1) name of the first author, publication year, and study location; 2) category of study design; 3) patient characteristics, such as information regarding the diagnosis, sample size, age, sex, duration of enrollment, proportions of patients with lung SCC or ACC in each study, and clinical stages of NSCLC; 4) cutoff values for TSR; 5) outcomes reported; and 6) confounding factors that were adjusted. The Newcastle-Ottawa Scale [28] was used for study quality evaluation based on three domains, including patients group selection, between-group comparability, and outcome determination. This scale were with a score band of 1–9, of which 9 indicates the highest study quality.

### Statistical Analyses

HRs and their corresponding 95% confidence intervals (CIs) were chosen as the general measure for the relationship between TSR and survival of patients with NSCLC. Data of HRs were extracted directly from the original stuides, and stand errors (SEs) of HRs were calculated based on 95% CIs or *p* values reported in the original papers. Data of HRs were further logarithmically transformed to stabilize variance and normalized the distribution [27]. The heterogeneity evaluation was achieved by the Cochrane’s Q test and calculation of I^2^ statistic [29]. A significant heterogeneity was considered if I^2^ > 50%. The outcome data was combined with a random-effect model by incorporating the between-study heterogeneity [27]. Sensitivity analyses by sequentially excluding one dataset a time, were performed to evaluate possible influence of certain study on the outcome [30]. Further sensitivity analyses were also performed by limiting to studies with multivariate analyses and studies with the cutoff of TSR as 50%. Subgroup analyses according to the histopathological type of the cancer were also performed (SCC versus AC). Besides, univariate meta-regression analyses were also performed evaluate the correlations between proportions of patients with SCC and AC in each study with the survival outcomes of patients with NSCLC. The potential publication bias was assessed by visual examination for the symmetry of the funnel plots and the results of Egger’s regression asymmetry test [31]. A *p* value <0.05 indicates statistically significance. If high risk of publication bias was determined, a “trim-and-fill” analysis was performed to estimate the influences of possible studies with negative findings on the meta-analysis outcome [27]. The RevMan (Version 5.1; Cochrane Collaboration, Oxford, United Kingdom) software was applied for the meta-analysis and statistics.

## Results

### Literature Search

**FIGURE 1:**
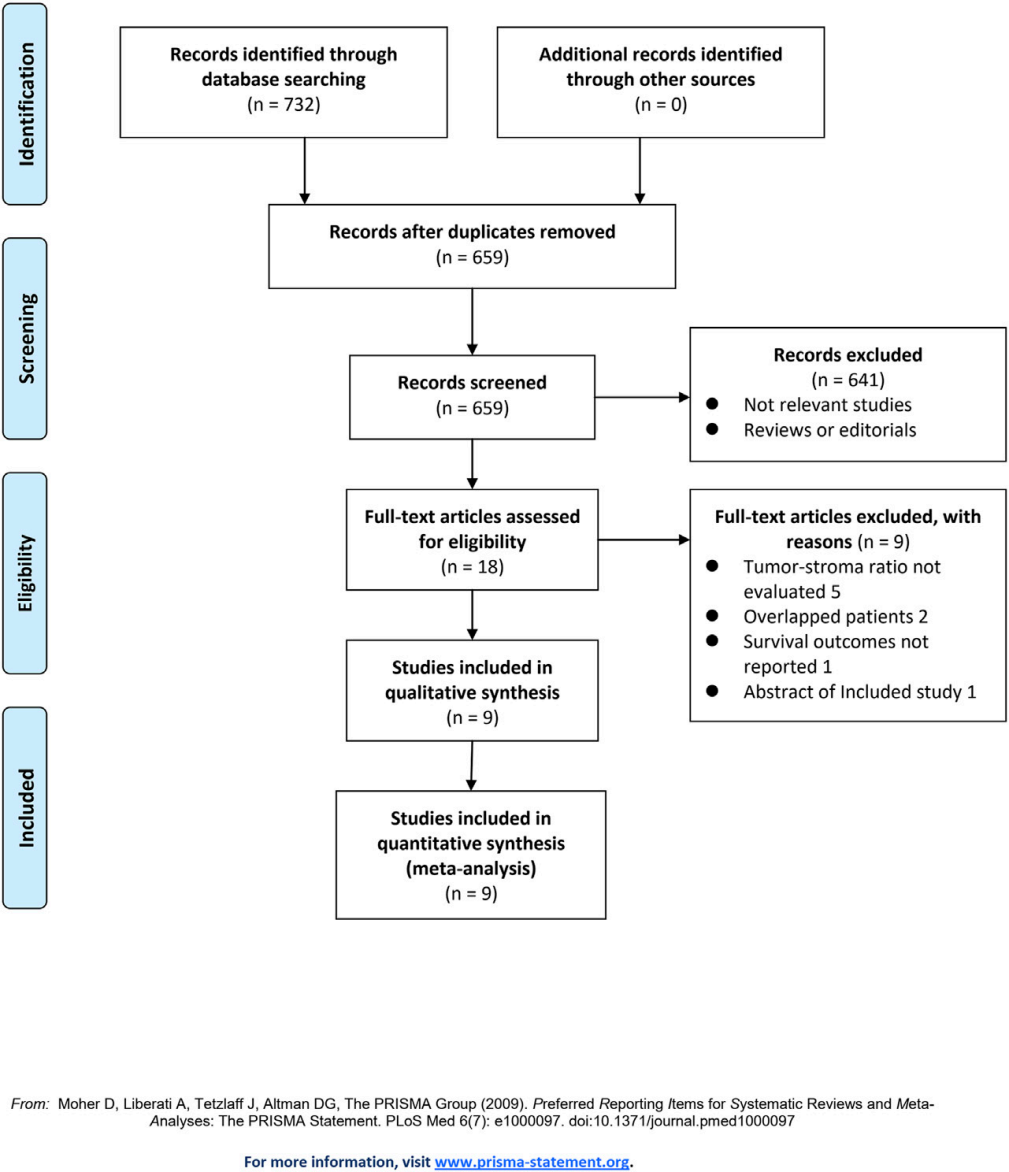
Flowchart of literature search.

Figure 1 shows the flowchart of database search and study identification. It was shown that a total of 659 articles were obtained after literature search in Medline, Embase, and Web of Science databases, while 641 of them were excluded based on titles and abstracts primarily because they were irrelevant studies. Then, nine of the 18 potentially relevant studies were further excluded based on full-text review, according to the reasons listed in Figure 1, and the other nine studies were finally included [14–22].

### Study Characteristics and Quality Evaluation

Table 1 shows the summarized characteristics of the included studies. These nine studies were all designed as retrospective cohort studies including patients with NSCLC from China, Japan, Turkey, the Netherlands, and Sweden. As for the histopathological features, four of them included patients with overall NSCLC [14, 15, 17, 19], and the remaining five included patients with lung squamous cell carcinoma (SCC) [16, 20–22] and adenocarcinoma (AC) [18, 22], respectively. The sample size for the studies varied between 76 and 520. The mean ages of the patients varied between 59 and 71 years, with the proportions of males ranging from 51 to 95%. Most of the studies included patients with stage I–III NSCLC, expect for one study which included patients with stage I–IV patients [20]. For five of the included studies [14, 15, 17, 19, 21], a TSR cutoff of 50% was used to define stroma-rich and stroma-poor NSCLC. Outcome of RFS was reported in seven studies [15–21], and OS was reported in eight studies [14–19, 21, 22]. In two of the included studies [16, 22], univariate analyses were performed to evaluate the association between TSR and survival outcome, while in the other seven studies [14, 15, 17–21], multivariate analyses were applied with the adjustment of potential factors such as age, sex, smoking, tumor size, stage, grade, and adjuvant therapy. Table 2 shows the summarized details of study quality evaluation. The NOS scores were six to nine for these studies, which reflected the generally good study quality.

**TABLE 1:** Characteristics of the included studies.

Study	Country	Design	Patient characteristics	Duration	Sample size	Mean age	Men	SCC	AC	Stage	Cutoff for TSR	Outcomes reported	Variables adjusted	NOS
						Years	%	%	%					
Wang 2013	China	RC	Patients with NSCLC underwent surgical resection	2000–2007	73	59	68.5	47.9	45.2	I–III	50%	OS	Age, sex, smoking, tumor size, histopathological type, lymphatic metastasis, differentiation grade, and tumor stage	8
Zhang 2015	China	RC	Patients with NSCLC underwent surgical resection	2007–2009	404	60	73	41.6	58.4	I–III	50%	RFS and OS	Age, sex, smoking, tumor size, histopathological type, differentiation grade, and tumor stage	8
Gürel 2016	Turkey	RC	Patients with lung SCC underwent surgical resection	NR	76	63	94.7	100.0	0.0	I–III	25%	RFS and OS	None	6
Xi 2017	China	RC	Patients with NSCLC underwent surgical resection	2007–2009	261	60	63.2	69.7	30.3	I–III	50%	RFS and OS	Age, sex, smoking, tumor size, histopathological type, lymphatic metastasis, differentiation grade, tumor stage, and adjuvant therapy	8
Ichikawa 2018	Japan	RC	Patients with lung AC underwent surgical resection	1999–2003	127	66	55.1	0.0	100.0	I–III	10%	RFS and OS	Age, sex, smoking, tumor size, nodal involvement, lymphatic permeation, vascular invasion, and pleural invasion	8
Zhang 2019	China	RC	Patients with NSCLC underwent surgical resection	2015–2017	520	60	73.1	41.5	58.5	I–III	50%	RFS and OS	Age, sex, smoking, tumor size, histopathological type, differentiation grade, and tumor stage	8
Koike 2020	Japan	RC	Patients with peripheral lung SCC underwent surgical resection	2002–2015	135	71	88.1	100.0	0.0	I–IV	33%	RFS	Age, sex, smoking, tumor size, nodal involvement, lymphatic permeation, vascular invasion, pleural invasion, and postoperative therapy	9
Smit 2020	the Netherlands	RC	Patients with lung SCC underwent surgical resection	2000–2018	174	66	81	100.0	0.0	I–III	50%	RFS and OS	Age, sex, tumor stage, and adjuvant therapy	8
Micke 2021-SCC	Sweden	RC	Patients with lung SCC underwent surgical resection	1987–2004	90	68	57.8	100.0	0.0	I–III	Median	OS	None	8
Micke 2021-AC	Sweden	RC	Patients with lung AC underwent surgical resection	1987–2004	171	66.7	44.3	0.0	100.0	I–III	Median	OS	None	8

TSR, tumor-stroma ratio; NOS, Newcastle-Ottawa Scale; RC, retrospective cohort; NSCLC, non-small cell lung cancer; SCC, squamous cell carcinoma; AC, adenocarcinoma; NR, not reported; RFS, recurrence-free survival; OS, overall survival.

**TABLE 2:** Details of study quality evaluation via the Newcastle-Ottawa scale.

Study	Representativeness of the exposed cohort	Selection of the non-exposed cohort	Ascertainment of exposure	Outcome not present at baseline	Control for age and sex	Control for other confounding factors	Assessment of outcome	Enough long follow-up duration	Adequacy of follow-up of cohorts	Total
Wang 2013	0	1	1	1	1	1	1	1	1	8
Zhang 2015	0	1	1	1	1	1	1	1	1	8
Gürel 2016	0	1	1	1	0	0	1	1	1	6
Xi 2017	0	1	1	1	1	1	1	1	1	8
Ichikawa 2018	0	1	1	1	1	1	1	1	1	8
Zhang 2019	0	1	1	1	1	1	1	1	1	8
Koike 2020	1	1	1	1	1	1	1	1	1	9
Smit 2020	0	1	1	1	1	1	1	1	1	8
Micke 2021	0	1	1	1	0	0	1	1	1	6

### TSR and RFS in NSCLC

**FIGURE 2:**
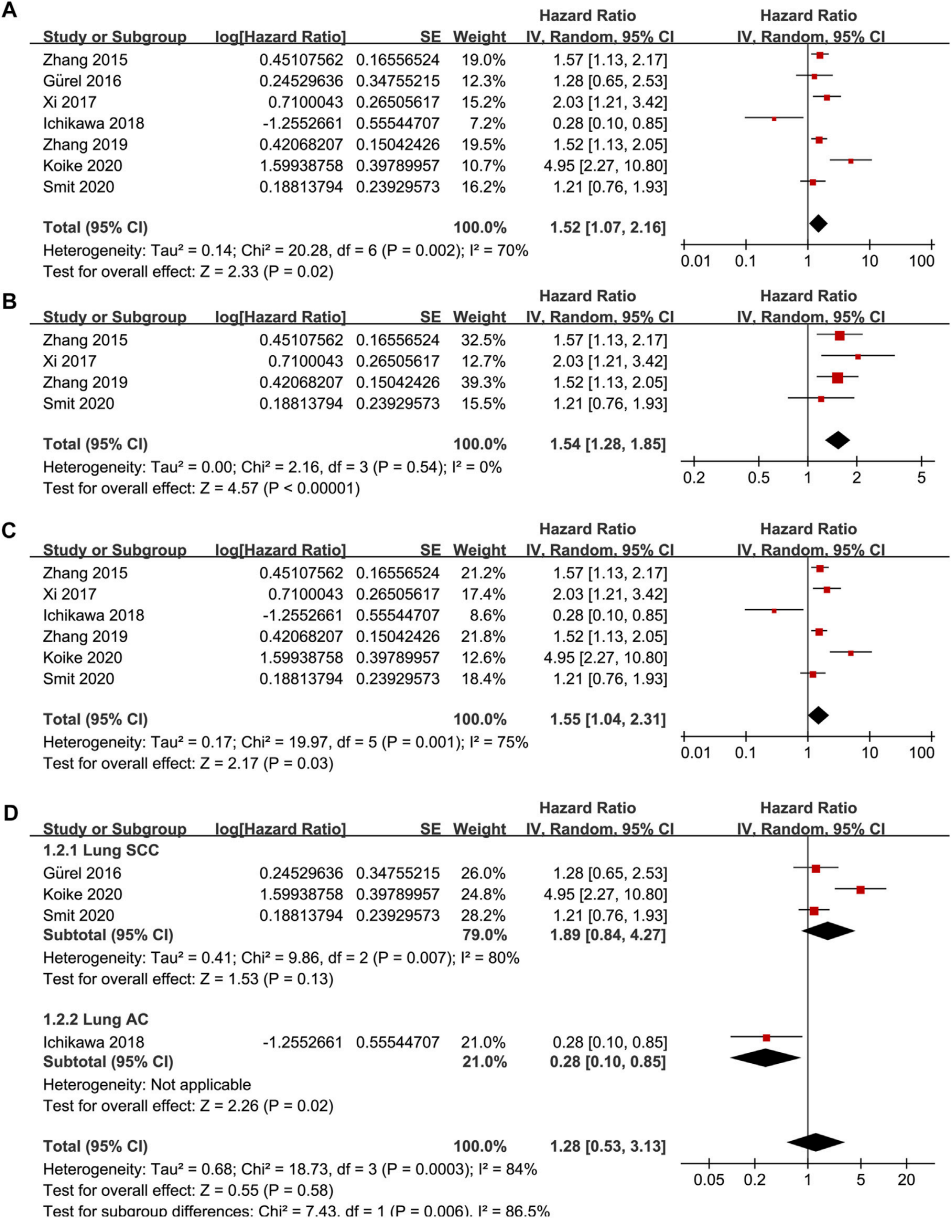
Forest plots for the meta-analysis of the association between TSR and RFS in patients with NSCLC; **(A)**, overall results for the meta-analysis; **(B)**, sensitivity analysis limiting to studies with TSR cutoff of 50%; **(C)**, sensitivity analysis limiting to studies with multivariate analyses; and **(D)**, subgroup analysis according to the histopathological type of the cancer; The effect size of each study is proportional to the statistical weight. The diamond indicates the overall summary estimate for the analysis; the width of the diamond represents the 95% CI.

Seven studies [15–21] reported the association between TSR and RFS in patients with NSCLC. Pooled results showed that compared to patients with stroma-poor NSCLC, those with stroma-rich NSCLC were associated with worse RFS (HR = 1.52, 95% CI: 1.07 to 2.16, *p* = 0.02; Figure 2A) with significant heterogeneity (I^2^ = 70%). Sensitivity analysis by excluding one study at a time did not significantly affect the result (HR: 1.37–1.68, *p* all <0.05). Further sensitivity analyses showed consistent results in studies with TSR cutoff of 50% (HR: 1.54, 94% CI: 1.28 to 1.85, *p* < 0.001; I^2^ = 0%; Figure 2B) and in studies with multivariate analyses (HR: 1.55, 95% CI: 1.04 to 2.31, *p* = 0.03, I^2^ = 75%; Figure 2C). Subgroup analyses suggested that higher content of tumor stroma may be non-significantly associated with poor RFS in patients with lung SCC (three datasets, HR: 1.89, 95% CI: 0.84 to 4.27, *p* = 0.13), but may be associated with better RFS in patients with lung AC (one dataset, HR: 0.28, 95% CI: 0.10 to 0.85, *p* = 0.02; *p* for subgroup difference = 0.006, Figure 2D). In addition, univariate meta-regression analyses showed that a higher proportion of patients with SCC was correlated with a higher HR for RFS (Coefficient = 0.012, *p* = 0.03; Figure 4A; Table 3), while a higher proportion of patients with AC was correlated with a lower HR for RFS (Coefficient = −0.012, *p* = 0.03; Figure 4B; Table 3).

**TABLE 3:** Results of univariate meta-regression analyses according to the proportions of patients with SCC and AC in each included study.

RFS			
Covariate	Coefficient	95% CI	*p*
SCC (%)	0.012	0.002 to 0.022	0.03
AC (%)	−0.012	−0.002 to −0.022	0.03
OS			
Covariate	Coefficient	95% CI	*p*
SCC (%)	0.014	0.003 to 0.025	0.02
AC (%)	−0.013	−0.001 to −0.025	0.04

AC, adenocarcinoma; CI, confidence interval; OS, overall survival; RFS, recurrence-free survival; SCC, squamous cell carcinoma.

### TSR and OS in NSCLC

**FIGURE 3:**
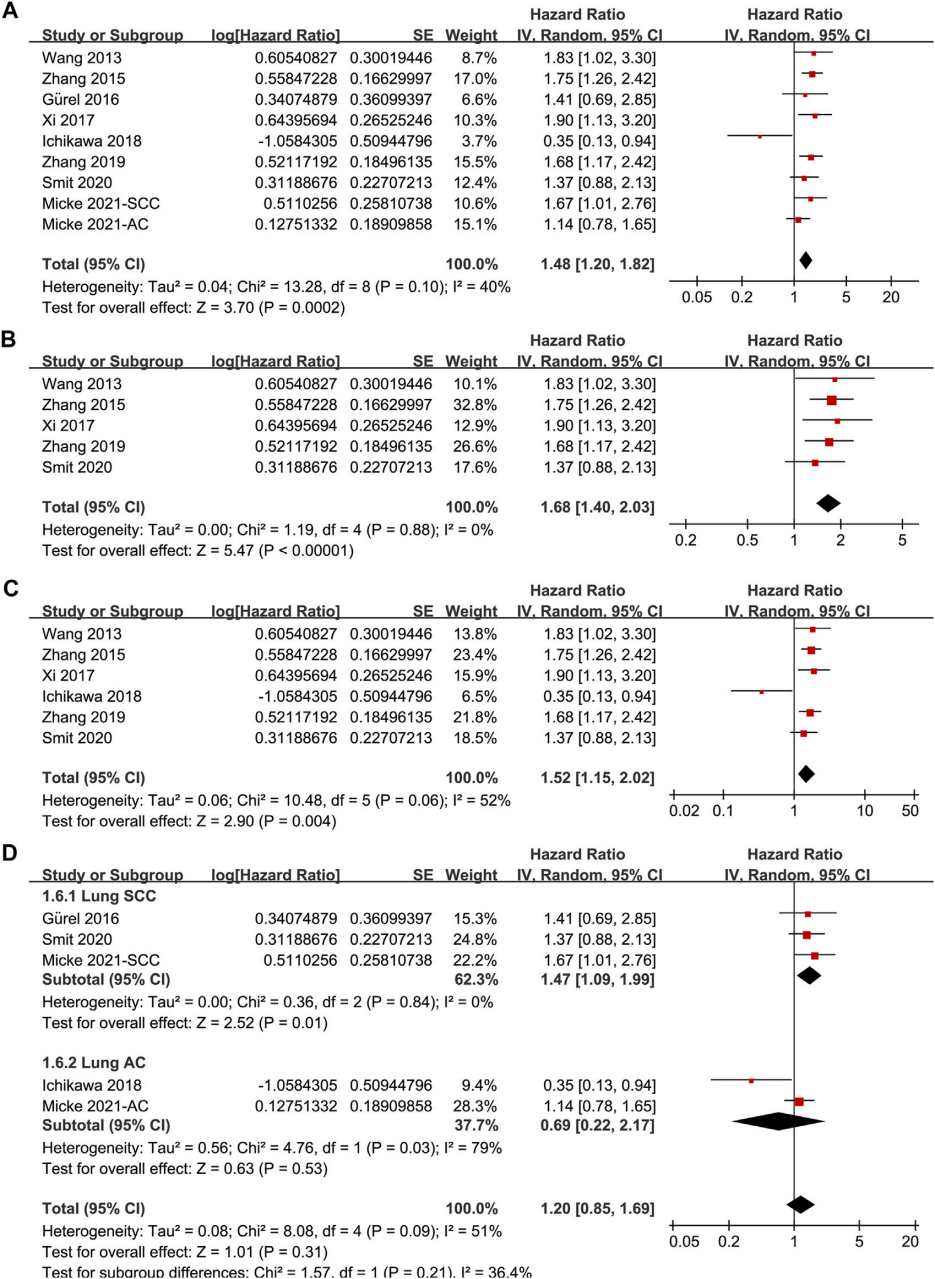
Forest plots for the meta-analysis of the association between TSR and OS in patients with NSCLC; **(A)**, overall results for the meta-analysis; **(B)**, sensitivity analysis limiting to studies with TSR cutoff of 50%; **(C)**, sensitivity analysis limiting to studies with multivariate analyses; and **(D)**, subgroup analysis according to the histopathological type of the cancer; The effect size of each study is proportional to the statistical weight. The diamond indicates the overall summary estimate for the analysis; the width of the diamond represents the 95% CI.

**FIGURE 4:**
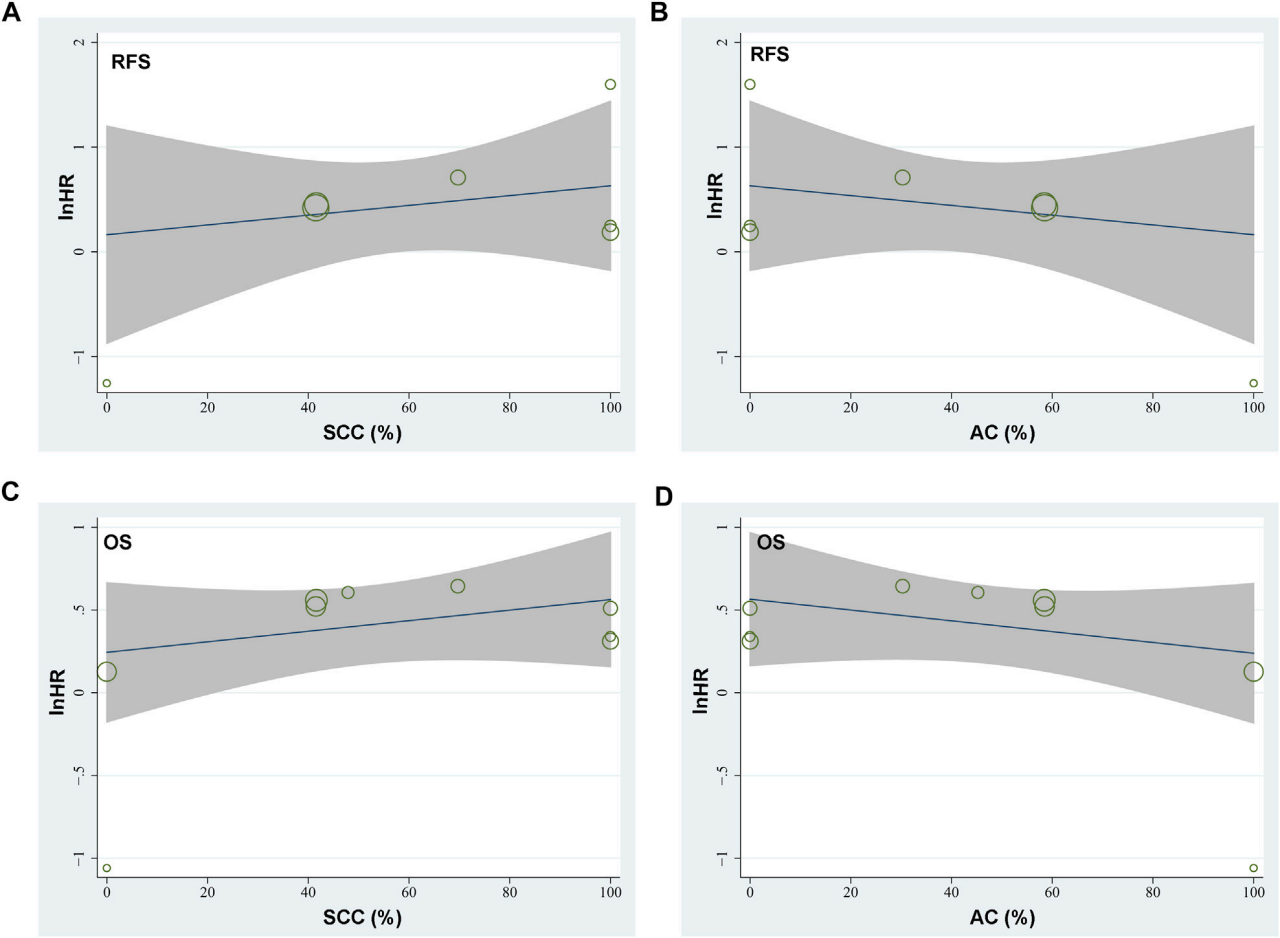
Graphic results of meta-regression analysis showing the correlations between proportions of patients with lung SCC and AC in each study with the logarithmically transformed HR (lnHR) for RFS and OS in patients with NSCLC. **(A)**, correlation between proportions of patients with lung SCC and lnHR for RFS; **(B)**, correlation between proportions of patients with lung AC and lnHR for RFS; **(C)**, correlation between proportions of patients with lung SCC and lnHR for OS; and **(D)**, correlation between proportions of patients with lung AC and lnHR for OS. The horizontal axis indicates the proportions of patients with lung SCC or AC, and the vertical axis indicates the lnHR values for RFS and OS. Each circle indicates an included dataset with the area of the circle indicates the weight of the study.

Since one of the included studies reported the association between TSR and OS in patients with lung SCC and AC separately [22], these datasets were independently included in the meta-analysis. Accordingly, eight studies [14–19, 21, 22] including nine datasets were available for the association between TSR and OS of NSCLC. Pooled results showed that patients with stroma-rich NSCLC had worse OS compared to those with stroma-poor tumor (HR = 1.48, 95% CI: 1.20 to 1.82, *p* < 0.001; Figure 3A) with moderate heterogeneity (I^2^ = 40%). Consistent results were retrieved by sensitivity analyses excluding one study at a time (HR: 1.42–1.56, *p* all <0.05), and limiting to studies with TSR cutoff of 50% (HR: 1.68, 94% CI: 1.40 to 2.03, *p* < 0.001; I^2^ = 0%; Figure 3B) and studies with multivariate analyses only (HR: 1.52, 95% CI: 1.15 to 2.02, *p* = 0.004, I^2^ = 52%; Figure 3C). Subgroup analyses suggested that higher content of tumor stroma was associated with poor OS in patients with lung SCC (three datasets, HR: 1.47, 95% CI: 1.09 to 1.99, *p* = 0.01), but may be non-significantly associated with better OS in patients with lung AC (two datasets, HR: 0.69, 95% CI: 0.22 to 2.17, *p* = 0.53; *p* for subgroup difference = 0.21, Figure 3D). Further univariate meta-regression analyses showed that a higher proportion of patients with SCC in each study was correlated with a higher HR for OS (Coefficient = 0.014, *p* = 0.02; Figure 4C; Table 3), while a higher proportion of patients with AC was correlated with a lower HR for OS (Coefficient = -0.013, *p* = 0.04; Figure 4D; Table 3).

### Publication Bias

**FIGURE 5:**
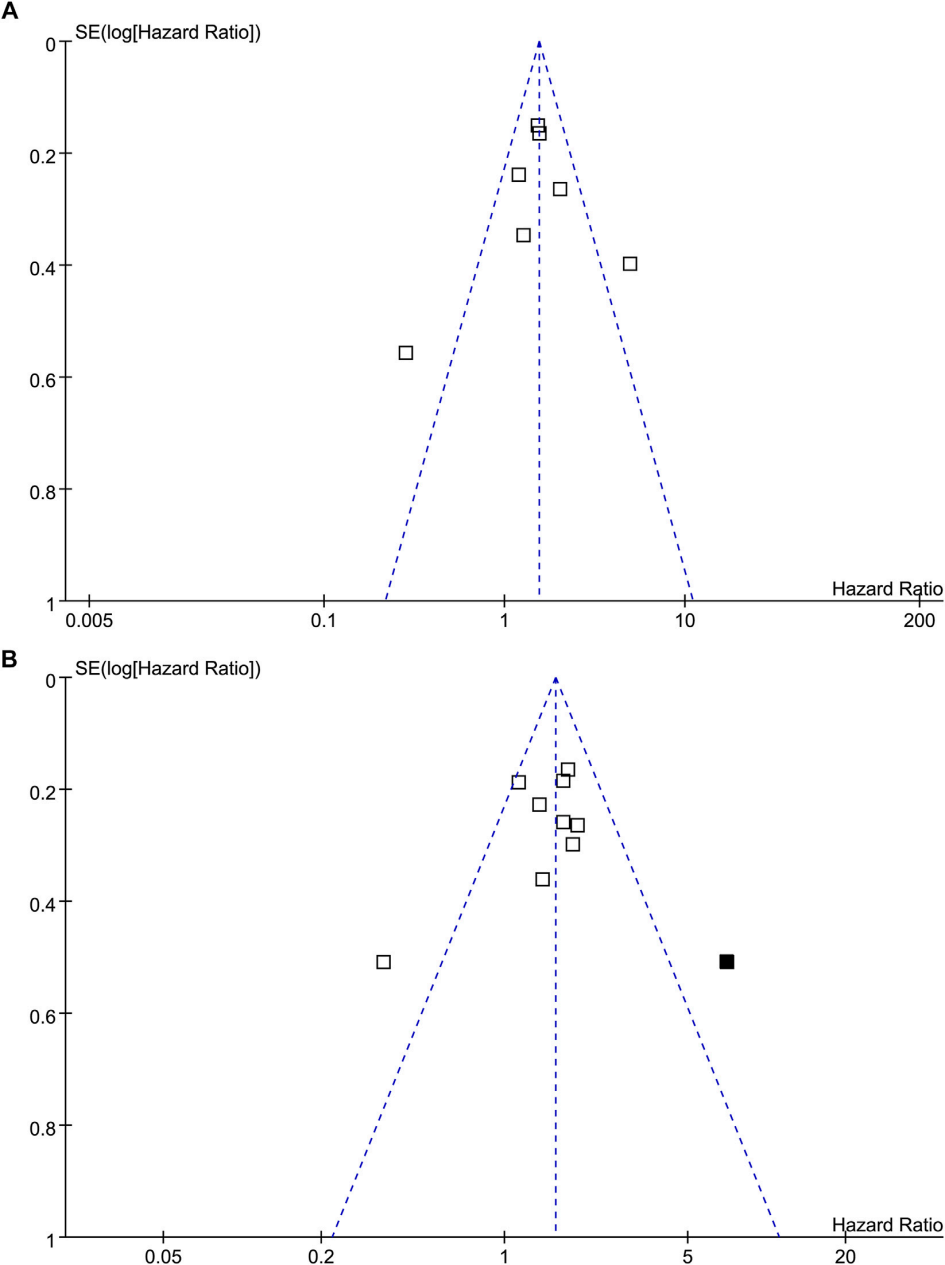
Funnel plots for the publication bias of the meta-analysis of the association between TSR and survival in patients with NSCLC; **(A)**, funnel plots for the outcome of RFS; and **(B)**, funnel plots with trim-and-fill analysis for the outcome of OS (black square indicates hypothesized study to achieve the symmetry of the funnel plots).

The funnel plots for the meta-analysis of the association between TSR with RFS and OS were shown in Figures 5A,B. The plots for RFS were symmetrical on visual inspection, suggesting low risk of publication bias. Results of Egger’s regression tests also suggested low risks of publication biases (*p* = 0.289; Figure 5A). The funnel plots for OS were asymmetrical, suggesting potential risk of publication bias. We therefore performed a trim-and-fill analysis. As shown in Figure 5B, incorporating the hypothesized study achieved symmetry of the funnel plots, and the results of the meta-analysis remained significant after including this hypothesized study (HR: 1.57, 95% CI: 1.21 to 2.02, *p* < 0.001, I^2^ = 60%).

## Discussion

In this meta-analysis, by combining the results of available cohort studies, overall results of the meta-analysis showed that compared to patients with stroma-poor (high TSR) tumor, patients with stroma-rich (low TSR) NSCLC were associated with significantly worse survival outcomes, as evidenced by RFS and OS. Sensitivity analysis showed that the results of the meta-analysis were not primarily driven by either of the included studies, indicating the robustness of the finding. Further sensitivity analyses showed consistent results in studies with TSR cutoff of 50% and in studies with multivariate analyses. Interestingly, results of subgroup analyses and meta-regression analyses suggested that the relative proportion of patients with SCC/AC in the included NSCLC patients may be an important determinant for the association between TSR and survival in NSCLC. Stroma richness may be a predictor of poor survival in patients with lung SCC, but a predictor of better survival in patients with lung AC. Taken together, current evidence from retrospective studies suggested that tumor TSR is a prognostic predictor of survival in patients with NSCLC, and the association between TSR and survival outcomes may be different between patients with lung SCC and AC.

As far as we know, this is the first meta-analysis evaluating the association between TSR in primary tumor and survival outcomes in patients with NSCLC. A few methodological strengths should be indicated before the results of the meta-analysis are interpreted. Firstly, we applied extensive literature retrieval and strict inclusion criteria during the study. Accordingly, the up-to-date literatures regarding the prognostic role of TSR in NSCLC were obtained. Besides, the stability of the finding was confirmed by performance of multiple sensitivity analyses. Results of the “leave-one-out” sensitivity analyses showed that the significance of the finding was not primarily driven by either one of the included studies. Sensitivity analysis limited to studies with multivariate analysis indicated that the association between TSR and survival of patients with NSCLC may be independent of potential confounding factors such as age, sex, smoking status of the patients, and size, stage, and grade of the tumor. Finally, exploring subgroup and meta-regression analysis was performed to explore the potential influence tumor histological type on the association. Although limited datasets were available for each subgroup, results of the subgroup and meta-regression analyses suggest that the association between high content of tumor stroma and poor survival were mainly observed in studies including patients with lung SCC, while for patients with lung AC, high content of tumor stroma may be a predictor of favorable survival outcomes.

The TSR, also known as the tumor stroma percentage, is measured on traditional hematoxylin and eosin (H&E)-stained paraffin sections at the invasive tumor front. Assessment of TSR has been conducted using routine HE stained glass slides and can be performed within a few minutes [32]. However, a standardized method for TSR remains to be determined and current methods for measuring of TSR varies and may be difficult to compare or reproduce. Aiming to standardize the method of assessment of TSR, van Pelt et al. [32] have recently introduced recommendations for the assessment of TSR. For scoring of TSR they recommended to consider the most deeply invasive part of the primary tumor. Areas with the highest amount of stroma are selected at low magnification (objective × 2.5 or × 5); then a stromal area which has tumor islands/cells present at all edges of the selected field is scored at a higher magnification (objective × 10). In cases of heterogeneity, the highest percentage of stroma is selected. Pelt et al. [32] further suggested using 50% as a cutoff value for dividing tumors as having low or high stromal content. Interestingly, these recommendations [32] were approved in substantial of the included stuides with NSCLC patients using HE-stained sections [14, 15, 17, 19, 21], and therefore they can be proposed as a standard method for the evaluation of TSR in daily practice.

The mechanisms underlying the potential role of components of tumor stoma in the progression of NSCLC are likely to be multifactorial [10]. For example, cancer-associated fibroblasts (CAFs), as a major component of cancer stroma, could promote tumor proliferation, invasion and metastasis and induce angiogenesis via the production and secretion of various cytokines and growth factors [33]. Besides, remodeling of the extracellular matrix by degrading proteases is also shown to be involved in the metastasis of NSCLC, which accounts for >70% of deaths in these patients [34]. Currently, tumor microenvironment, such as stroma of tumor has been involved in the pathogenesis and progression of NSCLC [8, 9]. However, index of tumour microenvironment or stroma has not been integrated in the risk stratification and treatment determination in patients with NSCLC. Compared to genetic or immune prognostic markers, TSR could be obtained by conventional pathological analysis with a microscope, which is simple, inexpensive, effective, and feasible in real-world clinical practice [35]. Moreover, treatments targeting tumour stroma and other components of tumor microenvironment may be effective and promising [36, 37]. Once therapy targeting tumor stroma becomes critical, measuring of TSR may be helpful to identify patients with optimal therapy response in patients with NSCLC. Taken together, although these results should be validated in large-scale prospective cohort studies, results of the meta-analysis suggested that TSR may become a useful prognostic predictor for the survival of patients with NSCLC.

Results of the subgroup and meta-regression analyses showed that the relative proportion of patients with SCC/AC in the included NSCLC patients may be an important determinant for the association between TSR and survival in NSCLC. Stroma richness may be a predictor of poor survival in patients with lung SCC, but a predictor of better survival in patients with lung AC. However, it should be noted that since limited number of datasets were included for each subgroup, the results should be considered to be exploratory and the interpretation should be very cautious. The potential reasons for the results are still unknown. Interestingly, previous studies showed that lung SCC and AC expression subtypes demonstrated significant differences in tumor immune landscape [23], suggesting potential differences in immune host response between lung SCC and AC. In addition, subsequent studies showed that types of tumor immune microenvironment, including programmed death-ligand 1 (PD-L1) expression had diverse impact on survival of patients with lung SCC and AC [24, 25]. Since immune cells and cytokines are important components of tumor stroma that critically take part in the process of cancer progression, differences of lung SCC and AC in expressions of tumor immune landscape may at least partly explain the potential different prognostic role of tumor stroma in patients with lung SCC and AC.

Our study has limitations. Firstly, studies available for the meta-analysis were retrospective, which may be confounded by the recall or selection biases. Therefore, prospective cohort studies are needed for validation. Secondly, the optimal cutoff of TSR for defining of stroma-rich and stroma-poor NSCLC remains to be determined. In addition, since this is a meta-analysis based on data of study level, we were unable to determine whether the prognostic role of TSR on survival of NSCLC could be affected by patient or tumor characteristics, such as age, ethnicity, and comorbidities of the patients, grade and stage of the tumor, concurrent anticancer treatments, and especially the histological type of NSCLC. Meta-analysis based on individual-patient data may be considered for further evaluation of the possible different association between TSR and survival outcomes between patients with lung SCC and AC. Finally, although the methods for TSR analysis among the included studies were easy and based on HE staining slides only, personal subjectivity during the process of TSR analysis could still affect the results. A standardized protocol for the measuring and analyzing of TSR remains to be determined and validated.

In conclusion, current evidence from retrospective studies suggested that tumor TSR is a prognostic predictor of survival in patients with NSCLC, and the association between TSR and survival outcomes may be different between patients with lung SCC and AC. Further large-scale prospective studies are needed to validate these findings, and standard protocols and optimal cut-off values for TSR need to be developed.

## Data Availability

The original contributions presented in the study are included in the article/supplementary material, further inquiries can be directed to the corresponding author.

## Abbreviations

AC, adenocarcinoma; CAF, cancer-associated fibroblast; NSCLC, non-small cell lung cancer; OS, overall survival; PD-L1, programmed death-ligand 1; RFS, recurrence-free survival; SCC, squamous cell carcinoma; TSR, tumor-stroma ratio.
